# Use of Cement Mortar Incorporating Superabsorbent Polymer as a Passive Fire-Protective Layer

**DOI:** 10.3390/polym14235266

**Published:** 2022-12-02

**Authors:** Sittisak Jamnam, Gritsada Sua-iam, Buchit Maho, Satharat Pianfuengfoo, Manote Sappakittipakorn, Hexin Zhang, Suchart Limkatanyu, Piti Sukontasukkul

**Affiliations:** 1 Construction and Building Materials Research Center, Department of Civil Engineering, King Mongkut’s University of Technology North Bangkok, Bangkok 10800, Thailand; 2 Department of Civil Engineering, Faculty of Engineering, Rajamangala University of Technology Phra Nakhon, Bangkok 10300, Thailand; 3 School of Engineering and the Built Environment, Edinburgh Napier University, Edinburgh EH11 4BN, Scotland, UK; 4 Department of Civil Engineering, Faculty of Engineering, Prince of Songkla University, Hat Yai Campus, Songkla 90110, Thailand

**Keywords:** superabsorbent polymers, cement plastering mortar, passive fire protection layer, compressive strength loss, moisture mitigation

## Abstract

Concrete structures, when exposed to fire or high temperatures for a certain time, could suffer partial damage or complete structural failure. Passive fire-protective coating materials are an alternative way to prevent or delay damage to concrete structures resulting from fire. Superabsorbent polymer (SP) is a synthetic material known for its ability to absorb and retain a large volume of water within itself. With this unique property, the SP exhibits great potential for use as a passive fire protection material. Although several studies have been carried out to investigate the effect of SP as a surface coating material for fire protection, very few have been investigated on the potential use of SP mixed with mortar as a passive fire-protective layer. The objective of this study is to introduce the use of SP in plastering mortar as a fire-protective layer for concrete subjected to temperatures up to 800 °C. This study is divided into two parts: (1) investigating the properties of cement mortar mixed with SP at 0.5% (CONC/SP-0.5) and 1.0% (CONC/SP-1.0) by weight of cement, and (2) investigating the potential use of SP mortar as a plastering layer for concrete subject to high temperatures. The experimental results showed that the density and compressive strength of SP mortar decreases with increasing SP dosages. From the heat exposure results, SP mortar exhibited lower strength loss due to the ability to mitigate moisture through its interconnected pore system. As for the use of SP mortar as a plastering layer, the results demonstrated the concrete specimen plastered with SP mortar had a lower temperature at the interface and core than that plastered with plain mortar. This led to a reduced strength loss of 20.5% for CONC/SP-0.5 and 17.2% for CONC/SP-1.0.

## 1. Introduction

Concrete structures may be exposed to fire incidents, which could lead to property damage and, in some cases, fatalities. There are numerous ways to prevent or reduce fire risk in buildings through governmental fire protection regulations [1] such as zoning (commercial vs. residential), standoff distance, the use of non-combustible interior decoration, etc. However, in cases where preventive measures fail and fire has already broken out, the use of excellent fire-resistant construction materials becomes essential.

Theoretically, concrete is a non-combustible material composed of cement and aggregates that when chemically mixed create an inert material with low thermal conductivity. Thus, the concrete itself cannot be set on fire, which is crucial for fire safety design. However, upon exposure to fire or high temperatures for a certain period, changes in the microstructure of concrete, due to a buildup of internal pressures, could lead to material disintegration (from expansion and cracking), a loss in load-carrying capacity and eventually, complete structural failure [2,3,4,5,6,7]. In a fire event, the chemical composition and physical structure of concrete change dramatically. At temperatures above 110 °C, dehydration occurs through the release of chemically bonded water from the calcium silicate hydrate. Internal stresses are increased by this dehydration as well as the thermal expansion of the aggregate. At about 530 °C, calcium hydroxide, one of the most significant components in cement paste, dissociates and causes the concrete to shrink [3]. When exposed to high temperatures between 300 and 800°C, concrete’s strength and Young’s modulus decrease dramatically, internal structures deteriorate, and microdefects appear on the micro- and mesoscales [7], which causes concrete to lose most of its compressive strength (at around 800 °C) [1]. To delay the process of disintegration, passive fire protection coating materials are normally applied, sprayed, or plastered on the surface of the structural members. The general requirements for these materials include low combustibility, low thermal conductivity, and a low rate of flame spread and smoke development. Gypsum board, calcium silicate, ceramic, rock, glass wool, or intumescent coatings are some examples [8]. Commercially available passive fire protection materials include cementitious coating mortar (i.e., perlite cement), fire retarder (ammonium polyphosphate), intumescent paints, fiber garment wrapping (i.e., aramid fiber), polymer-modified bitumen, etc. [9,10,11].

Cementitious materials are widely accepted for passive fire protection because of their cost effectiveness, low thermal conductivity, and non-combustibility. Cementitious materials are also compatible and work well with different kinds of property enhancement additives or materials on both micro- and macro-structure levels, for example, carbon nanotube [6,12], graphene oxide [13,14,15], polymeric materials [16,17,18,19], phase change materials [20,21,22,23], fibers [24,25,26,27,28,29], mineral materials [30,31,32], crumb rubber [33,34,35], etc. Especially in the case of polymeric material such as superabsorbent polymer (SP), during the past decade there has been a growing interest in the application of SPs in cement-based materials [36,37,38].

Superabsorbent polymer is a synthetic polymer material in hydrophilic groups, which can absorb a large amount of water, swell to a larger size, and retain the water. Superabsorbent polymers can generally absorb 100–1000 times more water than their own volume depending on the type. They are generally used as medical materials, diapers, and sanitary napkins, as well as plugging materials. Besides the ability to absorb large amounts of water, once dried they are capable of reabsorbing similar volumes of water. The potential uses of SP to enhance the properties of cement material have been reported by several researchers, for example, the improvement in rheological properties of fresh concrete, the mitigation of autogenous and plastic shrinkage, the reduction in drying shrinkage cracking, and the prevention of water leakage [39]. Superabsorbent polymers have proven to be effective in preventing self-desiccation and autogenous shrinkage in concrete with low water-to-binder ratios [39,40,41]. Superabsorbent polymers can be added to concrete in two ways: dry SPs require a specific amount of time to absorb water during the mixing process, and some pre-saturated SPs can even desorb some of the water during the mixing process [42]. In practice, the powdery pre-blend of all the dry ingredients of a concrete or mortar is batched into dry SPs and then swells during the mixing process after the addition of water and dissolved or liquid chemical admixtures [43].

Superabsorbent polymers in concrete were originally designed for internal curing to reduce autogenous shrinkage, emulating pre-saturated porous lightweight aggregates with tiny water reservoirs dispersed throughout the matrix [44,45,46]. The potential use of SPs as an internal curing agent in concrete and mortar is being investigated by researchers. The results show that SPs delay internal relative humidity drop and mitigate autogenous shrinkage at an early age [44]. The performance of the internal curing largely depends on the amount, particle size, and absorbed water of the SP [45]. However, due to the detrimental impact on the pore structure of cement paste, the compressive strength and splitting tensile strength of concrete decreases as the SP content increases, especially at higher water-to-binder ratios [46]. In addition, the inclusion of SP is unlikely to have a negative impact on the long-term strength of mortars [47,48].

The effectiveness of SPs in enhancing concrete durability was studied by Manzur et al. [49], who reported the increase in compressive strength and the decrease in both water and chloride permeability for SP concrete. Superabsorbent polymer can greatly improve the carbonation and chloride penetration resistance of concrete [50]. When pre-absorbed SP is combined with the removal of internal curing water from the mixing water, the carbonation resistance and chloride erosion resistance of concrete are significantly improved. Increased SP content also increases crack sealing performance [51]. Similarly, concrete mixed with SPs can improve both the frost resistance and thermal insulation properties of concrete [52].

In the case of fire protection, SP has been used as a fire retardant agent to spray directly or coat over the surface of structures [53,54]. However, for SP to be effective, it must be in a spray with water to achieve a fully moist condition. As for the application of SP with cement mortar related to fire protection, there is only one related study by Lura and Terrasi [55]. Therefore, the objective of this study is to contribute to the knowledge base related to the potential use of SP in cement mortar as a passive fire retardant. Our study is divided into two parts: (1) SP particles mixed with cement mortar to investigate the change in properties such as compressive strength, density, and loss of strength when exposed to heat; (2) SP mortar used as a plastering mortar to coat the concrete core to investigate its potential use as a fire retardant.

## 2. Research Methodology

The experimental series was divided into 2 stages. Stage 1 aimed to investigate the properties of cement plastering mortar mixed with SP in two different proportions. Stage 2 aimed to investigate the ability of plastering mortar as a passive fire resistant material.

### 2.1. Materials

Materials used in this study consisted of:

- Portland cement Type I (OPC) with properties in accordance with ASTM C150 [56]. It has a specific gravity of 3.15 and a specific surface area of 0.35 m^2^/g.
- Fine aggregate, which was natural river sand with specific gravity of 2.47 and fineness modulus of 3.08. The gradation conformed to ASTM C33 [57] with a maximum particle size smaller than 4.75 mm.
- Tap water with pH between 6 and 7.
- Polycarboxylate-based superplasticizer with properties according to ASTM C494 [58].
- Superabsorbent polymer (SP), sodium polyacrylate type, with particle size ranging from 297 to 1000 μm, maximum water absorption rate of 200 to 350 times the mass of water, and density of about 1.22 g/cm^3^. The properties are shown in Table 1.

### 2.2. Mix Proportion and Specimen Preparation

The specimens were prepared in two types according to the testing stage. For Stage 1, bare (unplastered)-type specimens were prepared and for Stage 2, plastered-type specimens were prepared.

#### 2.2.1. Bare-Type Specimen

The bare-type specimen consisted of three categories: (1) control specimens containing no SP (CON); (2) SP specimens containing SP at two different dosages: 0.5 and 1.0% by weight of cement (SP-0.5 and SP-1.0); (3) core concrete samples containing no SP (CONC) that were prepared for the plastered specimens (Section 2.2.2). For all specimen types, a water/cement (w/c) ratio of 0.50 and binder/fine aggregate (b/f) ratio of 0.37 were set. The details of the mix proportions are given in Table 2.

All bare specimens were prepared according to ASTM C305 [59]. The mixing process began with dry mixing all dry ingredients (OPC, fine aggregate, and SP) in a mixing machine at the speed of 60 rpm for 2 min, then about 90% of the liquid (water and superplasticizer) was added into the dry mix and the mixing continued with the speed of 120 rpm for 3 min. Finally, the rest of the liquid part (about 10%) was added to the fresh mix and the mixing continued for 3 min. The fresh mortar mixture was poured into cubical molds with dimensions of 50 × 50 × 50 mm and demolded after 24 h. The curing condition for plain specimens was divided into three conditions: air curing for 28 days, wet curing for 28 days, and wrap curing with plastic sheet for 28 days.

#### 2.2.2. Plastered Specimen

**Figure 1:**
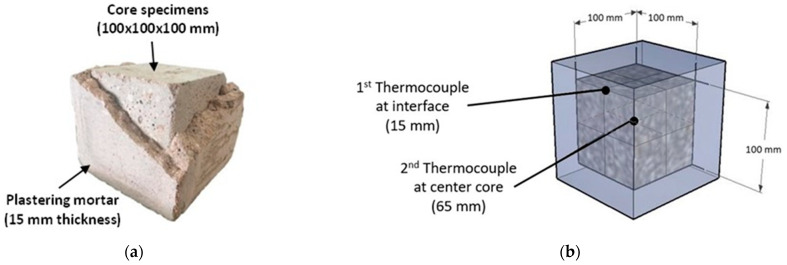
(**a**) Plastered specimen and (**b**) location of thermocouples.

For plastered specimens, the core concrete was prepared using a similar process as described in Section 2.2.1 in the form of cubical specimens with dimensions of 100 × 100 × 100 mm. The mix proportions of CONC shown in Table 3 provided an average compressive strength of 25.8 MPa cured under wrap condition at room temperature. After casting, the specimens were wrapped with plastic sheets for 28 days. After that, the specimens were plastered with CON, SP-0.5 and SP-1.0 mortars with the thickness of 15 mm and then wrapped for another 7 days before being subjected to heat exposure test (Figure 1).

### 2.3. Experimental Series

#### 2.3.1. Stage 1: Properties of Mortar-Incorporated SP

The experiment in this stage was carried out on bare specimens with the objective of investigating the properties of CON and SP mortars prior to and after being subjected to elevated temperatures before use as a plastering mortar in Stage 2. After 28 days of curing under different conditions, the density, compressive strength, and thermal properties of the specimens before being subjected to heat were investigated and used as reference values. The compressive strength was carried out in accordance with ASTM C109 using a 1500 kN universal testing machine (UTM). The mortar specimens were prepared in cubic form with dimensions of 50 × 50 × 50 mm. The specific heat capacity was carried out in accordance with ASTM C1269 using a differential scanning calorimeter. The samples from the specimens were taken in powder form from various parts of the specimen.

For the heating test, the specimens were placed in an electrical furnace chamber. The temperature was increased at a constant rate of 10 °C/minute until the target temperature was reached. Three target temperatures were set at 400 °C, 600 °C, and 800 °C. After reaching each target temperature, the specimens were left to cool down to room temperature before being removed from the chamber and tested. The density and compressive strength of the specimens before and after exposure to high temperature were conducted in accordance with ASTM C109 [60].

#### 2.3.2. Stage 2: Passive Fire Resistance of SP Plastering Mortar

The experiment in this stage was carried out on plastered specimens (CONC cubes covered with 15 mm thick SP mortars). After curing for 28 days, the plastered specimens were placed in an electrical furnace chamber and subjected to a constant temperature increase at a rate of 10°C/minute until the target temperature of 800 °C was reached, and then allowed to cool down to room temperature in the furnace. Two thermocouples were installed: one at the interface between plastering mortar and core specimen, and another at the center of the core specimen to measure the temperature-time history throughout the heating process (Figure 1). The results on both density and compressive strength loss after being subjected to elevated temperature were evaluated and discussed.

## 3. Results and Discussion

### 3.1. Stage 1: Properties of Mortar-Incorporated SP

#### 3.1.1. Surface Characteristics

**Figure 2:**
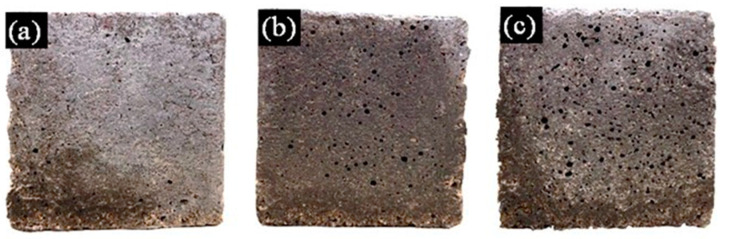
Surface characteristics of (**a**) CON, (**b**) SP-0.5, and (**c**) SP-1.0.

The surface characteristics were investigated on the bare-type specimens after 24 h of casting. The SP mortars appeared to have a darker shade than the control specimen due to the moisture that was absorbed by the SP particles during the mixing process that remained after the 24 h casting period (Figure 2). However, when considering the surface texture, the SP samples exhibited higher porosity than the control samples due to the effect of the SP particles in the mortar mix. The SP itself can not only absorb large amounts of water, but also has the ability to entrap water creating water bubbles during the mixing process similar to most liquid polymer substances [61]. The porosity was more obvious once the surface moisture evaporated from the SP particles and left behind empty voids. Comparing the three types, the number of surface pores of the SP-1.0 mortar was the highest, followed by SP-0.5 and then CON. Similar results were found by Al-Nasra et al. [62,63]; i.e., the addition of the SP content to the concrete mix created more voids in the mass of the concrete, which led to decreases in strength.

#### 3.1.2. Effect of SP on Dry Density

**Figure 3:**
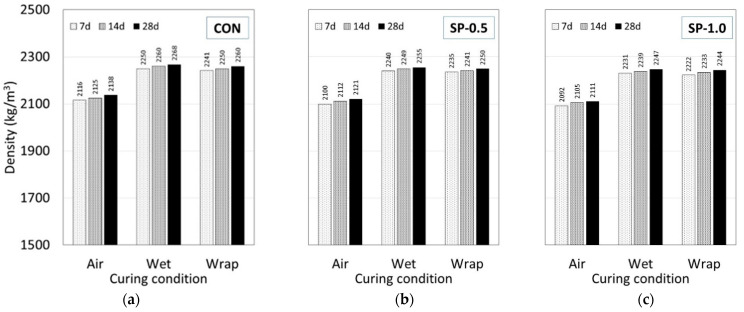
Dry density of (**a**) CON, (**b**) SP-0.5, and (**c**) SP-1.0 at different curing durations and conditions.

The dry density of the bare-type specimens subjected to different curing conditions is shown in Figure 3a–c. Based on the obtained results, the dry density was found to depend strongly on both curing condition and SP content. In the case of SP content, once the specimen was dried, the water that was absorbed by the SP during the mixing and trapped inside the specimen had evaporated leaving air voids, hence, lowering the dry density. For example, the dry density of CON, SP-0.5, and SP-1.0 was observed in the ranges of 2116–2268 kg/m^3^, 2100–2255 kg/m^3^ and 2092–2247 kg/m^3^, respectively. The addition of dry SP tends to increase air content and reduce the density, which is attributed to the relatively low specific gravity of the material and the increase in total water in the mix [42].

In the case of the curing condition, the density of the specimen cured under air-dry conditions exhibited the lowest density compared to the wrap and wet conditions. For the air-curing condition, the moisture can easily escape due to exposure to air; the lack of water caused an incomplete hydration process, which led to a highly porous microstructure and lower density [64]. For the wet curing condition, the specimens were submerged in water for a long period of time. This condition promoted a complete hydration process, which led to a much denser cement matrix and higher density. In the case of wrap curing, the effect of SP played an important role in absorbing and trapping moisture inside the SP particles; this created an internal curing condition and an ability to maintain the hydration process without external water supply, which yielded a dry density in a similar range to those subjected to wet curing [64,65,66]. Guan et al. [67] studied the effect of SP on the mechanical properties of cement and found that the SP can produce more hydration products than plain cement paste in the early stage due to the internal curing [50,68].

#### 3.1.3. Effect of SP on Compressive Strength

**Figure 4:**
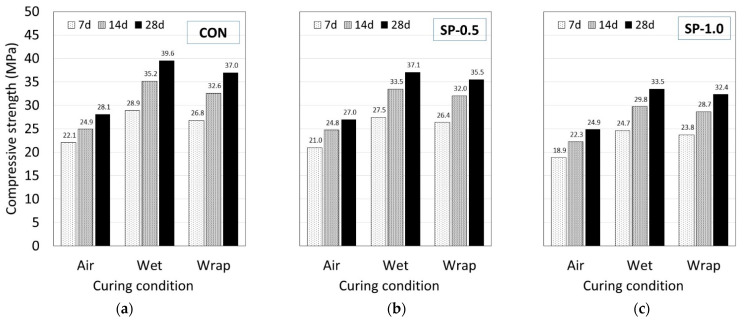
Compressive strength of (**a**) CON, (**b**) SP-0.5, and (**c**) SP-1.0 at different curing conditions.

The compressive strength of the CON and SP mortars tested at 7, 14, and 28 days under different curing conditions is shown in Figure 4a–c. Based on the obtained test results, regardless of the curing condition, the compressive strength was found to increase with increasing curing time. The compressive strength of CON, SP-0.5, and SP-1.0 was in the ranges of 22.1–39.6 MPa, 21.0–37.1 MPa, 18.9–33.5 MPa, respectively. Comparing between the three mortar types, both SP mortars exhibited lower compressive strength than CON. The strength also decreased with increasing SP content. As mentioned earlier, SP can absorb large amounts of water, thus creating a high volume of voids, which causes the strength of SP mortar to become lower than CON mortar [39,47,50,62,64].

Comparing between the different curing conditions, regardless of the mortar type, the specimens subjected to air curing exhibited the lowest compressive strength, followed by wrap and wet curing. The reason is similar to that mentioned in Section 3.1.2: the air curing condition allows moisture to evaporate easily; this creates an incomplete hydration reaction, which leads to high porosity and low compressive strength. In the case of wrap curing, when the water/cement ratio is sufficiently high, wrap curing prevents moisture from escaping, which helps maintain a continuous hydration reaction condition with the remaining water inside. The wet curing condition is an ideal case: where an unlimited amount of water is available during the curing period, the hydration reaction is much more complete, which leads to a more mature cement matrix with fewer voids and high compressive strength.

**Figure 5:**
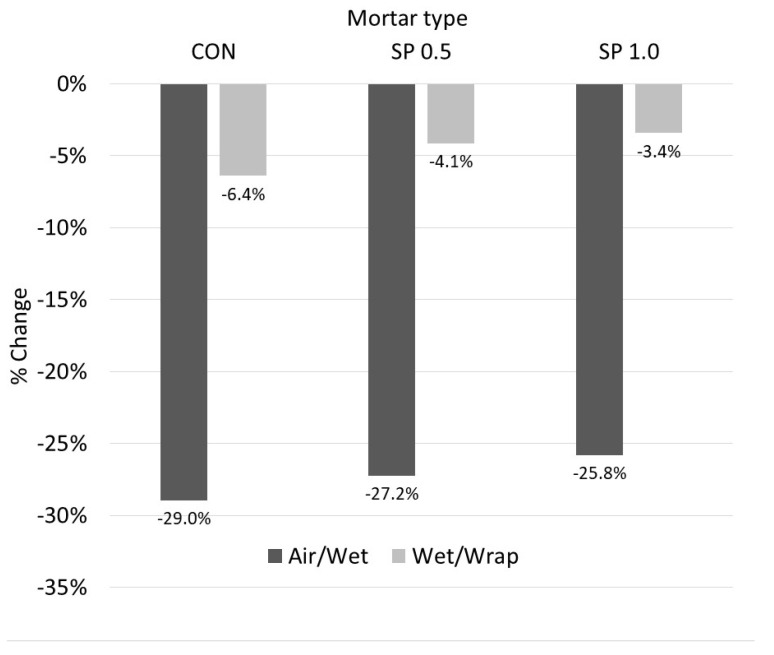
Percentage strength change vs. curing condition (28 days).

When comparing between each mortar type on the effect of the curing condition, since the ranges of the compressive strengths of each mortar type are not the same, the percentage difference in 28-day compressive strength was compared against the specimens subjected to wet curing (Figure 5). For both Air/Wet and Wrap/Wet curing conditions, the obtained results indicated that the percentage difference in the compressive strength of the SP mortars was lower than that of CON. Comparing between air and wet curing, percentage differences of −29.0%, −27.2%, and −25.8% were observed for CON, SP-0.5, and SP-1.0, respectively. Similarly, for wrap and wet curing, the largest percentage difference of −6.4% was observed in CON, followed by −4.1% in SP-0.5, and −3.4% in SP-1.0. The lower difference in SP mortar type indicated the ability of SP to absorb water and to create an internal curing condition in the environment where an external water supply was not available [41,69].

#### 3.1.4. Effect of SP on Thermal Properties

The thermal properties and specific heat capacity of hardened mortar specimens are given in Table 4. Based on the obtained test results, the addition of SP improved the thermal insulation properties but slightly reduced the specific heat capacity. The thermal conductivity values of CON, SP-0.5, and SP-1.0 were 1.95 W/m·K, 1.72 W/m·K, and 1.53 W/m·K, respectively. Moreover, the specific heat capacity values were 0.81 kJ/kg·K, 0.78 kJ/kg·K, and 0.75 kJ/kg·K for CON, SP-0.5, and SP-1.0, respectively. Comparing the three mortar types, both SP mortars exhibited lower thermal properties than CON; thermal conductivity decreased by 11.8% and 21.5%, whereas specific heat capacity decreased between 3.7 and 7.4% for SP-0.5 and SP-1.0, respectively. In general, thermal insulation increases with porosity [8]. The apparent difference in thermal conductivity was caused by the increased porosity of the SP mortar. In turn, the specimens’ thermal conductivity decreased as a result of the larger number of air gaps that impeded heat transfer. According to Al-Nasra et al. [52], employing sodium polyacrylate in the concrete mix can significantly improve the thermal insulation properties of concrete. As a material with good insulation properties, it can be used in the development of plaster or cement mortars as a passive fire protection solution in structures [8].

#### 3.1.5. Heat Exposure Test

The heating test was carried out on specimens that underwent wrap curing at 28 days. The specimens were weighed and recorded prior to being placed in the electrical heat chamber. After being subjected to heat, their weights were remeasured and recorded.

Table 5 shows the change in physical appearance of the mortar after being exposed to high temperatures at 400 °C, 600 °C, and 800 °C, and a shift in the surface color from gray to brown was observed with the increasing temperature. At 400 °C, no color change was observed; all mortar types had a natural grayish cement color. With the increase in temperature up to 600 °C, the color began to change to a more reddish shade due to the change in color of the silicate compound in the cement [64,70,71]. As the temperature increased to 800 °C, the color began to fade to a more brownish shade, which indicated the effect of burning on the specimen surface and the oxidation of the ferrous oxide in the aggregate [72,73].

**Figure 6:**
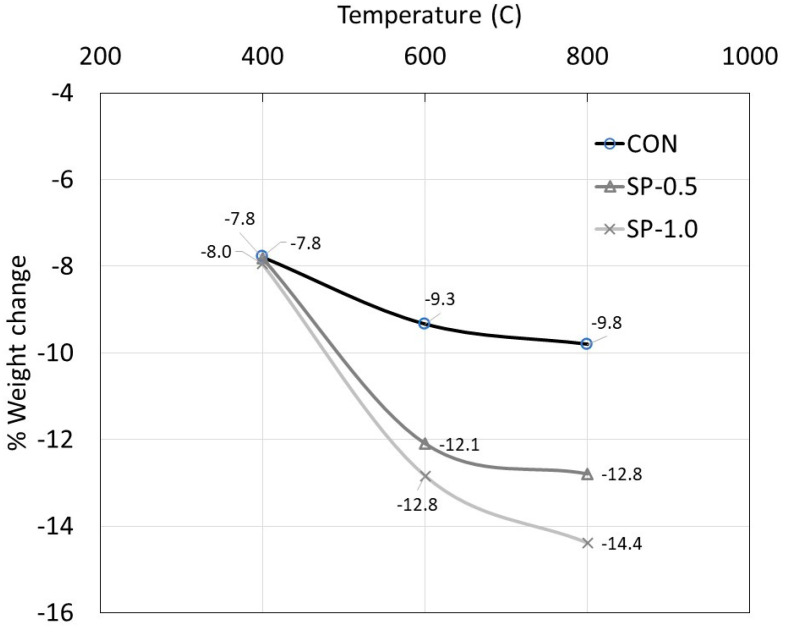
Weight change percentage after being heated.

Regarding weight change, the comparison was made in terms of weight change percentage before and after being subjected to heat. The results are shown in Figure 6. At 400 °C, a change in weight of about 7–8% was observed for all mortar types. There was almost no difference in weight change between each mortar type. Most of the moisture that evaporated out at 400 °C was suspected to come from capillaries and interconnected pores.

As the temperature increased to 600 °C and 800 °C, the weight change in CON samples began to slow down and a weight change percentage of 9.3–9.8% was observed. The moisture being driven out at this stage was believed to come from pores with much smaller diameters at the microstructural level such as gel pores. However, this is not the case for SP mortars where the weight change was still high (up to about 12.1–12.8% for SP-0.5 and 12.8–14.4% for SP-1.0). This was largely due to any evaporated moisture being absorbed by SP particles.

The compressive strength of bare specimens after exposure to heat is given in Table 6. Regardless of specimen type, the strength decreased with the increasing temperature. This is due to the presence of internal cracks inside the specimen resulting from the increasing internal pressures and stresses from the water being heated and the expansion of the fine aggregates [74,75,76]. Tufail et al. [77] indicated that the differential thermal expansion between cement paste and aggregates led to decreases in compressive strength under high temperature due to internal stresses.

**Figure 7:**
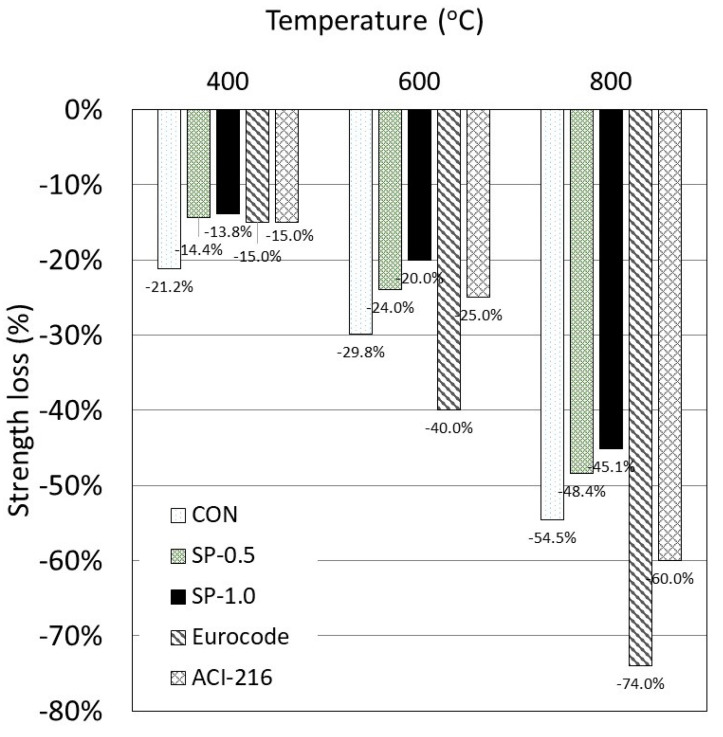
Percentage compressive strength loss of specimens after being exposed to heat.

To compare between each specimen type, since the maximum compressive strength of each type is different, the comparison was made based on the percentage change in the compressive strength of the samples exposed to different heat levels against those at room temperature (Figure 7). The results show the SP samples exhibited a lower strength loss percentage than the CON samples. The strength loss also decreased with increasing SP content. For example, at the highest temperature of 800°C, the strength loss percentage of CON, SP-0.5, and SP-1.0 was 54.5%, 48.4%, and 45.1%, respectively. The lower strength loss percentage in SP samples is partly due to the availability of moisture, which was absorbed inside the specimen and helped lower the internal temperature of the specimen during heat exposure (see Section 3.2). Another reason is because of the high porosity in the SP samples. The advantage of having high porosity is the ability to relieve internal pressure from boiling water during heat exposure. The network of highly interconnected pores allows water to escape from one pore to another during heating; this significantly reduces pressure and the risk of internal cracking [1,2,7].

For design purposes, the compressive strength reduction coefficient (SRC) (Table 6 and Figure 7) was compared to the results obtained using design curves prescribed in the Eurocode [78] and ACI 216 [79]. The experimental results revealed that the coefficient of strength reduction for the SP samples was similar to the design standard values, particularly at temperatures of 400 °C and 600 °C. At 800 °C, the coefficient from the design standard values is clearly underestimated according to the test results. When concrete is exposed to high temperatures, its compressive strength is unavoidably reduced, and concrete’s compressive strength significantly reduces at 800 °C [1]. As seen in Figure 7, when temperatures were increased to 800 °C, the strength of the bare specimens decreased more dramatically than that of the SP samples, but the SRC was higher than the design standard curve.

### 3.2. Stage 2: Passive Fire Resistance of SP Plastering Mortar

**Figure 8:**
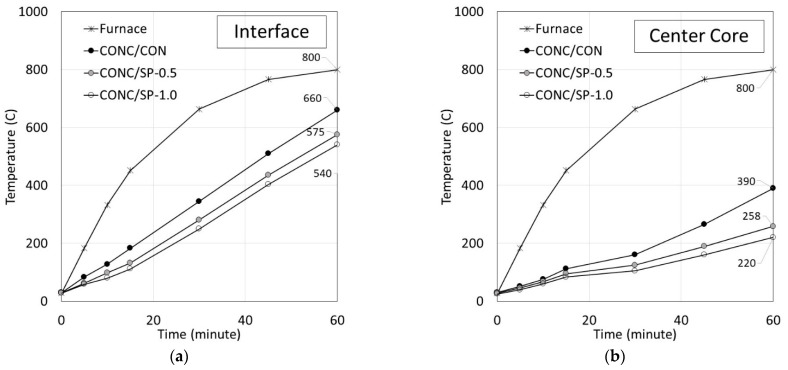
Temperature patterns at (**a**) Interface and (**b**) Center core.

The temperatures are measured at two locations: at the interface and center of the concrete core. The results shown in Figure 8a,b indicate the increase at both locations with the increasing oven temperature. The temperature at the interface between the plastering mortar and concrete core was higher than at the center of the core when compared at the same time. The CONC/CON exhibited the highest temperature followed by CONC/SP-0.5 and CONC/SP-1, respectively. When the oven temperature reached 800 °C, at the interface the highest temperatures of CONC/CON, CONC/SP-0.5, and CONC/SP-1 were observed as 660 °C, 570 °C, and 540 °C, respectively. Similarly, at the center core location, the highest temperatures of CONC/CON, CONC/SP-0.5, and CONC/SP-1 were observed as 390 °C, 258 °C, and 220 °C, respectively.

The lower temperature in samples plastered with SP (CONC/SP) was the direct result of the high moisture content in the SP plastering mortar. The moisture was absorbed by the SP particles during the mixing process and remained there during the heating test. The existence of moisture inside the plastering mortar helped slow down the heat transfer inside the specimen, as seen by the lower temperature at the interface and at the center core. The lower temperature also implies that the CONC/SP samples suffered less damage from heating than the CONC/CON samples.

After the heat exposure test, the samples were tested for compressive strength to investigate their strength loss and the effect of the SP plastering mortar. The results are given in Table 7. The specimens plastered with SP mortar clearly exhibited smaller strength loss than those plastered with CON. The CONC/CON suffered the highest strength loss of about 26.5% while the CONC/SP-0.5 and CONC/SP-1.0 exhibited smaller strength loss of 20.3% and 17.2%, respectively. This shows the ability of SP particles to absorb large amounts of moisture, mitigate pressure from boiled water, reduce internal damage, and lower compressive strength loss. In summary, due to the incorporation of SP as a passive fire protection material in a different way than in prior research [8], plastered concrete with increased fire-resistant mortars is a solution that achieves these protection requirements.

## 4. Conclusions

Based on the obtained experimental results, the following conclusions can be drawn.

The properties of SP mortar were found to depend strongly on the SP content and curing condition. The increase in SP content caused a decrease in dry density due to the expansion of the SP particles and their ability to trap air bubbles during the mixing process. The decrease in dry density also led to a decrease in compressive strength and thermal conductivity.

Under the different curing conditions, air curing exhibited the lowest strength followed by wrap and then wet curing. The moisture absorbed by SP particles helped promote internal curing for the SP mortars under wrap curing, causing them to have both dry density and compressive strength in a similar range as the SP mortars under moist curing conditions. After being exposed to high temperature, the loss of compressive strength in the SP mortars was lower than CON mortars.

In the case of plastering mortar, the SP mortar showed high potential for use as a fire-protection coating material as seen by the lower temperature at the interface and at the center core of the CONC/SP compared to CONC/CON specimens. The CONC/SP specimens also exhibited lower strength loss than CONC/CON specimens.

## Figures and Tables

**Table 1:** Properties of SP.

Structure	Dimension (Mesh)	Water Absorption Capacity (g/g)	Water Content (%)	pH	Density (kg/m^3^)
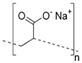	20–50	350	≥7	5.5–6.5	1220
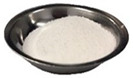			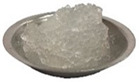		
Dry			Wet		

**Table 2:** Mixture proportions for bare specimens.

Designation	w/c	OPC (kg/m^3^)	Fine Aggregate (kg/m^3^)	Coarse Aggregate	Water (kg/m^3^)	Superplasticizer (%)	SP (%)
CON	0.5	550	1513	-	275	3.0	0
SP-0.5	0.5	550	1513	-	275	3.0	0.5
SP-1.0	0.5	550	1513	-	275	3.0	1.0
CONC	0.5	550	1100	2200	275	-	-

**Table 3:** Mixture proportions for plastered specimens.

Designation (a/b)	Mix Proportion for CONC (a)						Plastering Material (b)
	w/c	OPC (kg/m^3^)	Fine Aggregate (kg/m^3^)	Coarse Aggregate (kg/m^3^)	Water (kg/m^3^)	Superplasticizer (%)	
CONC/CON	0.5	550	1100	2200	275	3.0	CON
CONC/SP-0.5	0.5	550	1100	2200	275	3.0	SP-0.5
CONC/SP-1.0	0.5	550	1100	2200	275	3.0	SP-1.0

Note: a/b where a is the core concrete and b is plastering material.

**Table 4:** Thermal properties of specimens.

Designation	Thermal Conductivity (k-Value) (W/m·K)	Specific Heat Capacity (C-Value) (kJ/kg·K)
CON	1.95	0.81
SP-0.5	1.72	0.78
SP-1.0	1.53	0.75

**Table 5:** Surface characteristics of specimens after being subjected to heat.

Temperature (°C)	CON	SP-0.5	SP-1.0
400	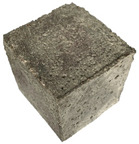	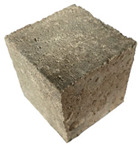	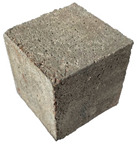
600	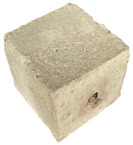	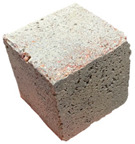	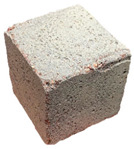
800	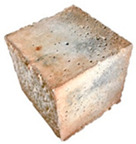	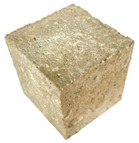	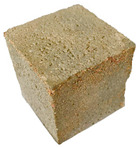

**Table 6:** Compressive strength of bare specimens after being exposed to heat.

Temperature (°C)	Compressive Strength (MPa)			Strength Reduction Coefficient (SRC)				
	CON	SP-0.5	SP-1.0	CON	SP-0.5	SP-1.0	Eurocode	ACI
Room	37.9	35.3	34.2	-	-	-	-	-
400 °C	29.9	30.2	29.5	0.788	0.856	0.862	0.850	0.850
600 °C	26.6	26.8	27.4	0.702	0.760	0.800	0.600	0.750
800 °C	17.2	18.2	18.8	0.455	0.516	0.549	0.260	0.400

Note: Strength reduction coefficient refers to the ratio between the compressive strength tested at high temperature and the compressive strength tested at room temperature.

**Table 7:** Compressive strength of plastered samples after heat exposure.

Specimen Type	Compressive Strength (MPa)	Strength Loss (%)	Remark
CONC	25.9	-	Room temperature
CONC/CON	18.9	−26.5%	800 °C Exposure
CONC/SP-0.5	20.5	−20.3%	800 °C Exposure
CONC/SP-1.0	21.3	−17.2%	800 °C Exposure

## Data Availability

Not applicable.
